# RateML: A Code Generation Tool for Brain Network Models

**DOI:** 10.3389/fnetp.2022.826345

**Published:** 2022-02-14

**Authors:** Michiel van der Vlag, Marmaduke Woodman, Jan Fousek, Sandra Diaz-Pier, Aarón Pérez Martín, Viktor Jirsa, Abigail Morrison

**Affiliations:** ^1^ Simulation and Data Lab Neuroscience, Institute for Advanced Simulation, Jülich Supercomputing Centre (JSC), Forschungszentrum Jülich GmbH, JARA, Jülich, Germany; ^2^ Institut de Neurosciences des Systèmes, Aix Marseille Université, Marseille, France; ^3^ Institute of Neuroscience and Medicine (INM-6) and Institute for Advanced Simulation (IAS-6) and JARA-Institute Brain, Jülich, Germany; ^4^ Computer Science 3–Software Engineering, RWTH Aachen University, Aachen, Germany

**Keywords:** brain network models, domain specific language, automatic code generation, high performance computing, simulation

## Abstract

Whole brain network models are now an established tool in scientific and clinical research, however their use in a larger workflow still adds significant informatics complexity. We propose a tool, RateML, that enables users to generate such models from a succinct declarative description, in which the mathematics of the model are described without specifying how their simulation should be implemented. RateML builds on NeuroML’s Low Entropy Model Specification (LEMS), an XML based language for specifying models of dynamical systems, allowing descriptions of neural mass and discretized neural field models, as implemented by the Virtual Brain (TVB) simulator: the end user describes their model’s mathematics once and generates and runs code for different languages, targeting both CPUs for fast single simulations and GPUs for parallel ensemble simulations. High performance parallel simulations are crucial for tuning many parameters of a model to empirical data such as functional magnetic resonance imaging (fMRI), with reasonable execution times on small or modest hardware resources. Specifically, while RateML can generate Python model code, it enables generation of Compute Unified Device Architecture C++ code for NVIDIA GPUs. When a CUDA implementation of a model is generated, a tailored model driver class is produced, enabling the user to tweak the driver by hand and perform the parameter sweep. The model and driver can be executed on any compute capable NVIDIA GPU with a high degree of parallelization, either locally or in a compute cluster environment. The results reported in this manuscript show that with the CUDA code generated by RateML, it is possible to explore thousands of parameter combinations with a single Graphics Processing Unit for different models, substantially reducing parameter exploration times and resource usage for the brain network models, in turn accelerating the research workflow itself. This provides a new tool to create efficient and broader parameter fitting workflows, support studies on larger cohorts, and derive more robust and statistically relevant conclusions about brain dynamics.

## 1 Introduction

Understanding the relationship between structure and function in the brain is a highly multidisciplinary endeavour; it requires scientists from different fields to develop and explore hypotheses based on both experimental data and the theoretical considerations from diverse scientific domains (Peyser et al., 2019). Because of this, simulation platforms have become essential tools to understand different states of the brain and promise, in the future, to provide a way of reproducing enough features of brain activity in order to better understand healthy brain states, diseases, aging, and development (Einevoll et al., 2019).

One particularly promising approach is whole-brain simulation based on non-invasive brain imaging techniques suitable for use in human studies (Lynn and Bassett, 2019). Functional and structural imaging modalities including Electroencephalography (EEG), Magnetoencephalography (MEG), Magnetic Resonance Imaging (MRI), and functional Magnetic Resonance Imaging (fMRI) allow researchers to capture characteristics of the brain primarily at a mesoscopic scale. The brain activity measured by such methods can be mathematically modelled and simulated using The Virtual Brain simulator (TVB; Sanzleon et al., 2013). Due to its ability to directly provide links between simulated outputs and experimental data, TVB is quickly gaining popularity in the scientific and clinical disciplines.

One of the strengths of such whole-scale brain simulation is the possibility of personalization of the model for a particular subject (Falcon et al., 2016; Bansal et al., 2018; Hashemi et al., 2020). This happens first on the level of structure—using person-specific connectivity and brain shape data, and second on the level of inferring the model parameters based on functional measurements of the subject at hand. The basic approach for personalising the simulated model behaviour entails finding the best fit between the numeric solution of the derivative equations, which determine the behaviour of the model, and the patient-specific functional empirical data (Deco et al., 2014). The differential equations representing a single neural mass prescribe the temporal evolution of multiple states, such as mean membrane potential or firing rate, as a function of initial conditions and parameters, such as the reversal potential and intra-mass coupling strength. To find a match between model and patient data, many model parameters need to be explored over potentially large ranges. Consequently, large parameter exploration are frequently carried out at high performance computing (HPC) centers.

Translating the set of differential equations into a concrete implementation is complex, as several factors can dramatically influence performance and correctness of the simulation. End users, such as clinicians or experimental neuroscientists, typically lack the background in programming necessary to implement a correct numeric implementation of their model and optimize it by exploring minor variations of the mathematics.

We therefore conclude that abstracting the modeling from the computational implementation, such that model descriptions can be automatically translated into correct and performant implementations (Blundell et al., 2018), would considerably aid these scientists to exploit the possibilities of whole-brain simulation. To this end, we have developed RateML, a modeling workflow tool that uncouples the specification of Neural Mass Models (NMMs) and Brain Network Models (BNMs) from their implementations as machine code for specific hardware. It is based on the existing domain specific language ‘Low Entropy Model Specification’ (LEMS; Cannon et al., 2014), which allows the user to enter declarative descriptions of model components in a concise XML representation. RateML enables users to generate brain models based on an XML format in which the generic features of rate-based neuron models can be addressed, without needing extensive knowledge of mathematical modelling or hardware implementation. In addition to providing code generation of the described models in Python, it is also possible to generate Compute Unified Device Architecture (CUDA) (NVIDIA et al., 2020) code in which variables of interest can be designated with a range for parameter exploration. The generated Python code can be directly executed within the TVB simulation framework, whereas the CUDA code has a separate driver module which is also generated before execution. The generated driver module enables the user to perform the explorations on any CUDA capable Graphics Processing Unit (GPU).

In this article we describe and benchmark the model generator RateML. In addition to the performance of the generated models, we investigate the maximum capacity for parameter sweeps and the scaling of the application on HPC. This article is structured as follows: after a summary of the state of the art in Section 2, we describe the model generator and the elements which are the building blocks for both the Python and CUDA models in Section 3. We then employ a use-case derived from a study on the impact of neuronal cascades on the causation of whole-brain functional dynamics at rest (Fox and Raichle, 2007) as a scaffold to demonstrate how to set up an existing model and validate it. In this study a parameter space exploration on two parameters of the Montbrió NMM is performed. Section 4 details the steps taken to express the Montbrió NMM in RateML. For the validation of the models generated by RateML, we reproduce the study’s parameter space exploration and analyse the results of this in Section 4.2. In Section 5, we examine the benchmark results for the Python and CUDA frameworks, comparing the Kuramoto (Kuramoto, 1975), Reduced Wong Wang (Wong and Wang, 2006) and Epileptor (Jirsa et al., 2014) models. From the benchmarks we observe that the application scales linearly with parameter space size and that it is memory bound. The performance increases with the parameter space size, indicating better data locality and decreasing memory latency.

With this work we provide a new modeling tool to the highly interdisciplinary community around brain research which bridges neuroscientific model descriptions and optimized software implementations. RateML opens new alternatives to better understand the effects of different parameters on models and large experimental data cohorts.

## 2 State of the Art

Computational models in neuroscience are becoming ever more complex. There is also an increasing desire to fully leverage new computing architectures to accelerate the simulation of brain dynamics at different scales (Furber et al., 2012; Abi Akar et al., 2019; van der Vlag et al., 2020). Code generation has become a popular approach to unburden users from manually creating models and to separate them from the underlying hardware and corresponding libraries, and indeed a multitude of modeling languages are available that simplify many aspects of brain simulation (Blundell et al., 2018). For example, NestML (Plotnikov et al., 2016) is a domain specific language for the NEST simulator which focuses on the description of point neuron models and synapses. NeuroML (Gleeson et al., 2010) is able to describe single cells and networks of cells, and NineML (Davison, 2013) focuses on networks of point neurons. Some simulators such as GeNN (Yavuz et al., 2016) and Brian (Stimberg et al., 2019) provide their own pipelines to transform abstract representations of models either in C or in Python and transform them into efficient executable code. Here, we focus on frameworks suitable for representing the dynamic variables of mesoscopic brain activity models.

One such framework mentioned by Blundell et al. (2018) is LEMS, a metalanguage designed to generate simulator-agnostic domain-specific languages (DSL) for graph-like networks (Cannon et al., 2014). Each node can have local dynamics described by ordinary differential equations, using the provided standardized structured descriptions. When the models are described in the LEMS XML format, a one-to-one mapping of these abstract components to the simulator specific functionality in the model file can be performed. The Mako templating library written in Python is well suited for such operations. Mako is an embedded Python language providing placeholders and logic to build a template from the ordered LEMS components. Mako is fast and supports Python control structures such as loops and conditionals, and code can be organized using callable blocks.

LEMS is a strong standard for the description of neural models and networks, used by a variety of simulators from the neuroscience community. It offers a set of generic building blocks, but currently, no domain specific language building on LEMS supports the whole variety of features required to express BNNs and NMNs. For example, if a user would like to perform large parameter explorations on models defined with the standard LEMS, she would need to rely on external software to coordinate the execution of parallel instances of the model on the target computing resources, also considering configuration and computing/memory access performance.

As an alternative to LEMS, PyRates is a Python framework that provides intuitive access to and modification of all mathematical operators in a graph (Gast et al., 2019). This enables a highly generic model definition. The aim of PyRates is to configure and simulate the model with only a few lines of code. Each model must be represented by a graph (circuit) of nodes and edges. The nodes represent the model units (for example, the cell populations) and the edges represent the information transfer between them. Circuits may be nested arbitrarily within other circuits, forming more complex sub-circuits. PyRates can, in principle, implement any kind of dynamical neural system. The resulting graph description can be then executed on CPUs, GPUs, or many node compute clusters.

To combine computational feasibility with biophysical interpretability, three mathematical operators are used to define the dynamics and transformations. The rate-to-potential operator (RPO) transforms synaptic inputs into average membrane potentials while the potential-to-rate operator (PRO) transforms the average membrane potential into an average firing rate output. The coupling operator (CO) transforms outgoing into incoming firing rates and is used to establish connections between populations; the weight and delay of such a connection are considered attributes of the corresponding edge. Individual network nodes consists of operators, which define scopes in which a set of equations and related variables are uniquely defined. The mathematical syntax closely follows the conventions used in Python. Vector and higher-dimensional variables may be used and it follows the conventions of NumPy (Harris et al., 2020).

For the actual simulation, the user can choose between two backend implementations. The first is the default NumPy backend which provides relatively fast simulations on a single CPU, or on multiple CPUs in combination with the Python distribution provided by Intel. The second is the Tensorflow 2.0 implementation, which makes use of dataflow graphs to run parallel simulation on CPUs or GPUs (Abadi et al., 2015). It can also apply vectorization, which reduces identical nodes to one vectorized node.

PyRates offers parallelization based on the inherent capabilities of Tensorflow. This generic approach has the downside that it does not optimize the parallelization depending on specific characteristics of the model to be executed.

Therefore, there is still a need for an automatic code generation framework that not only shares important qualities of the aforementioned tools, such as compact model specification and direct connection to simulation backends, but that is also able to support parameter sweep specification and highly optimized model-specific code generation.

## 3 The RateML Framework

In this manuscript we present RateML, a tool that enables users to generate BNNs and NMNs from a declarative description in which the mathematics of the model are described without specifying how their simulation should be implemented. Furthermore, RateML provides language features that permit parameter sweeps to be easily specified and deployed on high performance systems.

By building on LEMS, RateML joins a widely used standard which is in continuous development. RateML is able to automatically generate code which can be executed on CPUs and CUDA-compatible GPUs. RateML’s optimized CUDA backend takes full advantage of the device in order to maximize the occupancy based on the characteristics of the model like state variables and memory requirements. RateML also offers a Numba-based vectorized backend for execution on CPUs. In case of the latter, a generalized universal function using Numba’s guvectorize decorator is generated. Using this decorator, a pure Python function that operates over NumPy arrays can be compiled directly into machine code. This is as fast as a C implementation and it automatically uses features such as reduction, accumulation and broadcasting to efficiently implement the algorithm in question (Lam et al., 2015).

This section provides an overview of the implementation of the RateML framework which supports the definition of models and generation of both Python and CUDA code.

### 3.1 RateML Syntax

LEMS was originally designed to specify generic models of hybrid dynamical systems (Cannon et al., 2014). The LEMS language can be used to define the structure and dynamics of a wide range of biological models. The ComponentType building blocks can be defined as templates for model elements. Many of the ComponentTypes used in LEMS have a one-to-one mapping with the building blocks that make up the models for the TVB simulator. For instance, TVB has models which are defined by the dynamics of state variables represented by time derivatives, for which LEMS has placeholders. For these reasons, we adopted LEMS to create the code generation tool RateML, using the ComponentTypes as place holders to build and directly parse the NMMs dynamical models.

**FIGURE 1:**
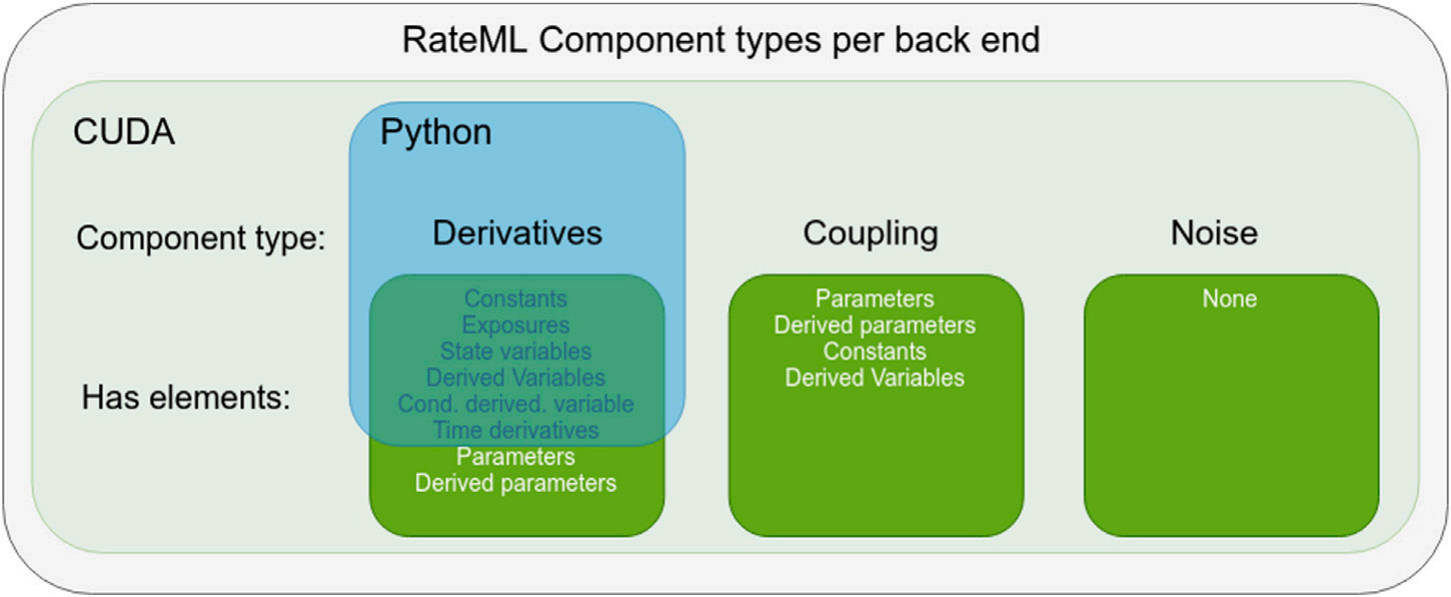
Overview of LEMS components adopted by RateML. The blue square indicates the ComponentType and the elements used to build Python models with a Numba kernel. The light green rectangle indicates the components types used to build CUDA models. The Python ComponentTypes are a subset of those used for CUDA.

Because not all ComponentTypes are necessary for the TVB models, RateML implements its syntax on top of a subset of components from LEMS. Figure 1 shows an overview of the adopted LEMS components which can be used to build BNMs and NMMs for TVB. The ComponentTypes defined for Python, which run in the native TVB simulator, are a subset of those defined for CUDA. Firstly, the ComponentTypes coupling and noise are excluded as building blocks, as they can be enabled by adding a single line of code when setting up the TVB simulator. Secondly, the elements Parameters and DerivedParameters of the ComponentType Derivatives are excluded, as these are needed for extensive parameter space exploration, for which the native TVB simulator is not designed.

Section 3.1.1 details the components that describe the differential equations. These components are used to build the Python Numba models and the CUDA models. Section 3.1.2 describes the components defining the coupling of the models for the CUDA implementation and Section 3.1.3 describes how stochastic integration is enabled. The Python models only require the definition of the time derivatives, while the CUDA models also require the parameter space exploration (PSE) to be configured and the coupling and stochastic integration of the time derivatives to be defined (if needed by the modeller).

An empty XML template called model_template.xml, can be found in the XMLmodels folder of RateML in the TVB repository^1^ and can be used as a blank template to start the construction of a model. The same folder hosts XML examples of the Kuramoto, Wong Wang, Epileptor, Montbrió, and Generic 2D oscillator models. Further documentation is also available in this repository.

#### 3.1.1 Derivatives


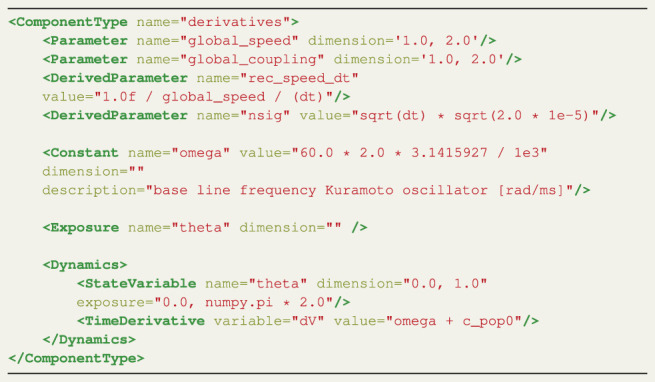


Listing 1 XML derivatives definition for the CUDA Kuramoto model.

The ComponentType Derivatives enables the specification of the NMM equations that define a model’s dynamical behaviour. It contains a number of elements which support the differential equations. An overview of all the elements of the Derivatives ComponentType are listed in Table 1.

**TABLE 1:** Overview of all the elements of the ComponentType *derivatives*.

Element	Generates	Has fields	Numba or CUDA	**Special**
Parameter	Parameter for sweeps	Name-Dimension	CUDA	
Derived Parameter	Expression for parameters	Name Value	CUDA	rec_speed_dt
Constants	Constant scalar variables of type float	Name Dimension Value Description	Both	nsig
Exposures	Simulation objects to monitor	Name Dimension	Both	
State Variables	Definition of state Variables and conditions	Name Dimension Exposure	Both	
Derived Variable	Temporary variables to support formulation of time derivatives	Name Value	Both	
Conditional derived Variables	If-else statements	Name	Both	
Case	Case with condition for if-else	Condition Value	Both	
Time Derivatives	The dynamic equation	Variable Value	Both	

The Parameter element defines the parameters targeted in the parameter sweeps. A Cartesian product is created over the parameter ranges, which generates all possible configurations of parameters. Each thread of the CUDA kernel takes a single parameter configuration and runs a TVB simulation. The resolution for each parameter, which determines how many threads are spawned, can be set on the command line (see Section 3.3).

There are two special DerivedParameters, rec_speed_dt and nsig. This DerivedParameter represents a unitary delay based on the transmission speed of the white fibers per integration unit. This convenient operation preprocesses the global_speed such that the CUDA kernel only has to perform a single multiplication instead of two divisions for the calculation of each input signal coupled to each node. The former sets the conduction speed to match TVB (no conduction delays if unset) and the latter is a multiplicative parameter to scale noise variance (see Section 3.1.2 and Section 3.1.3 for the coupling and stochastics components, respectively).

As a worked example, we will consider the implementation in RateML of the Kuramoto model (Kuramoto, 1975). The differential equation that defines the behavior of the Kuramoto model is

$$\frac{\theta_{n}}{dt}=\underset{A}{\underset{︸}{\omega_{n}}}+\underset{B}{\underset{︸}{k\overset{N}{\underset{p=1}{\sum }}C_{np}\;\sin\left(\theta_{p}\left(t-\tau_{np}\right)-\theta_{n}\left(t\right)\right)}}+\underset{C}{\underset{︸}{\eta_{n}\left(t\right)}},\;n=1,\ldots ,N$$ (1)

where *θ*_*n*_ denotes the phase of node *n* at time *t* (Cabral et al., 2011), *ω*_*n*_ is the intrinsic frequency of the node *n* on its limit cycle, *k* is the global coupling strength, *C*_*np*_ is the relative coupling strength, which is usually expressed as the weight of the connection between node *p* and node *n*, *τ*_*np*_ = *d*_*np*_/*v* is the delay between node *p* and node *n* and is calculated by dividing the distance between the nodes by the conduction speed *v*, and *η*_*n*_ is a Gaussian white noise term. The lengths and weights matrices are defined in a connectivity file outside of the XML file, which can be specified in the TVB simulation setup phase.

Eq 1 can be broken down into three terms, separated at the plus signs. Term *A* represents a constant contribution to the dynamics, the sum in term *B* defines the coupling, and term *C* defines the noise added to the model. These terms correspond to the three ComponentTypes in RateML, namely derivatives, coupling and noise.

The first ComponentType that needs to be defined is named derivatives. Listing 1 shows an XML file with all the elements and fields for the derivatives ComponentType. The elements in LEMS have to be used in an specific order. First, the elements used for the parameter sweeps are defined. In this case the global_speed and global_coupling are swept, which corresponds to the *v* and *k* terms as described above. The range for the parameter sweep is defined by the dimension field; in this example, both parameters will be swept between the values of 1.0 and 2.0. Note that the resolution is defined when the model is called on the command line, see Section 3.3). Next, two DerivedParameters named rec_speed_dt and nsig are defined. The DerivedParameter rec_speed_dt sets the conduction speed similar to the format used by TVB. This derived parameter is used to calculate the delay between the nodes which is the length between node n and p multiplied by rec_speed_dt. The lengths are specified in the connectivity matrix. The Parameter global_coupling reappears in the coupling ComponentType.

Having defined the sweeping parameters and the derived parameters, the constant *ω*_*n*_ (omega) is defined and its value set. Next, the dynamic variables which the user wishes to monitor are identified using the exposure element. In this example, as the Kuromoto model only has one dynamic variable, *θ*, only theta can be monitored.

Finally, the dynamics of the model are defined. The StateVariable element is used to identify *θ* (theta) as a dynamic variable; the range of the dimension field is used for its random initialization, in this case a value between 0.0 and 1.0, and the exposure field defines its boundaries. The exposure field has a different meaning than the Exposure element; these definitions are an inheritance from LEMS. In this example, *θ*, which represents the phase of the oscillation, is constrained to the range between 0.0 and 2*π*. The TimeDerivative element holds the equation for *θ*, here the sum of the constant omega and the result of the coupling defined by c_pop0, discussed below in Section 3.1.2. This corresponds to terms *A*+ *B* in Eq 1. The noise term does not need to be explicitly included in the TimeDerivative element and is discussed below in Section 3.1.3.

#### 3.1.2 Coupling

Term *B* is the part of Eq 1 that defines the coupling between the nodes. It is usually a sum of a function of the signals received by all other connected regions into each region. The coupling ComponentType gives the user freedom to define arbitrary coupling functions for the CUDA code generation; note that to identify this ComponentType, the name field must include the string ‘coupling’.

During the coupling computation the temporal properties of the brain network are taken into account. All nodes that are connected to the current node, the node for which the coupling is being calculated, are delayed before reaching it. This means that not the current states but states at previous timesteps of the connected nodes should be used to calculate the state of the current node. These previous states are fetched from memory. The computed delay, mentioned above in Section 3.1.1, serves as the timestep index to fetch the delayed states. Next to the temporal-also the spatial properties of the network are computed. The weight of the connected nodes, *C*_*np*_ in Eq 1 determines how strong the connection is.

Listing two shows a possible coupling relation of the Kuramoto model. The coupling elements have a similar keyword naming scheme to derivatives but differ slightly. The dynamic state variable for which the coupling relation needs to be computed, can be identified with the Parameter element. The Kuramoto model has only one state, thus the dimension, which is used to select the state, is set to ‘0’, because that is where the indexing starts. For models with multiple dynamic variables, the user is able to appoint any of these for the coupling function.

The delayed states of the connected nodes are stored in a user defined temporary variable (theta_p in the example), and can be used as a building block in the ComponentType for the coupling. This fetching of these delayed states, corresponds to the first part of the coupling term: *θ*_*p*_ (*t* − *τ*_*np*_), where *τ*_*np*_ is the delay, derived from the connectivity matrix.

Next, the DerivedParameter element defines a function that can be applied to the gathered result of all delayed nodes. In this case the gathered result is stored in c_pop0, which was previously used in the TimeDerivative element in the ComponentType derivatives defined in the section above. The function applied is a multiplication (which is implicit) with the global_coupling parameter, the target for the sweeps, corresponding to the multiplication by *k* in Eq 1.

In TVB, coupling components are composed of two functions: *pre* is applied before the summation over neighboring nodes, and *post* is applied after the summation. The DerivedVariable elements with the name pre or post are used to define the pre- and post synaptic coupling function. The pre-synaptic definition corresponds to the sin (*θ*_*p*_ (*t* − *τ*_*np*_) − *θ*_*n*_(*t*)) in term *B* of Eq 1. The model does not require post-synaptic activity.
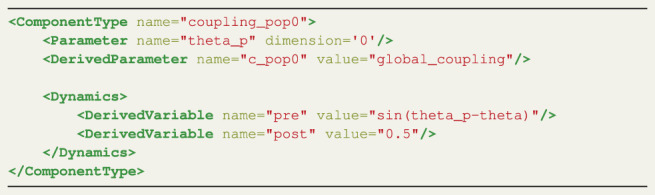


Listing 2 XML coupling definition for the CUDA Kuramoto model.

#### 3.1.3 Stochastics


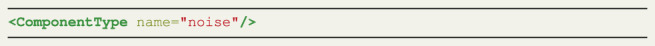


Listing 3 XML stochastics definition for the CUDA Kuramoto model.

To enable stochastic integration, a ComponentType with the name noise can be defined, see Listing 3. As discussed above, this is only implemented for the CUDA variant. The CUDA code generation makes use of the Curand library (NVIDIA, 2008) to add a normal distributed random value to the calculated derivatives. This corresponds to term *C* in Eq 1.

As mentioned in Section 3.1.1, if the derivatives ComponentType has a derived parameter with the name nsig, a noise amplification/attenuation is applied to the noise before it is added. In listing 1 an example of this derived parameter is shown on line 7. In this case the value for nsig is $\sqrt{dt}*\sqrt{2.0*1e^{-5}}$ , which acts as a multiplicative term to the noise term and is a commonly used attenuation in TVB.

### 3.2 XML to Model

**FIGURE 2:**
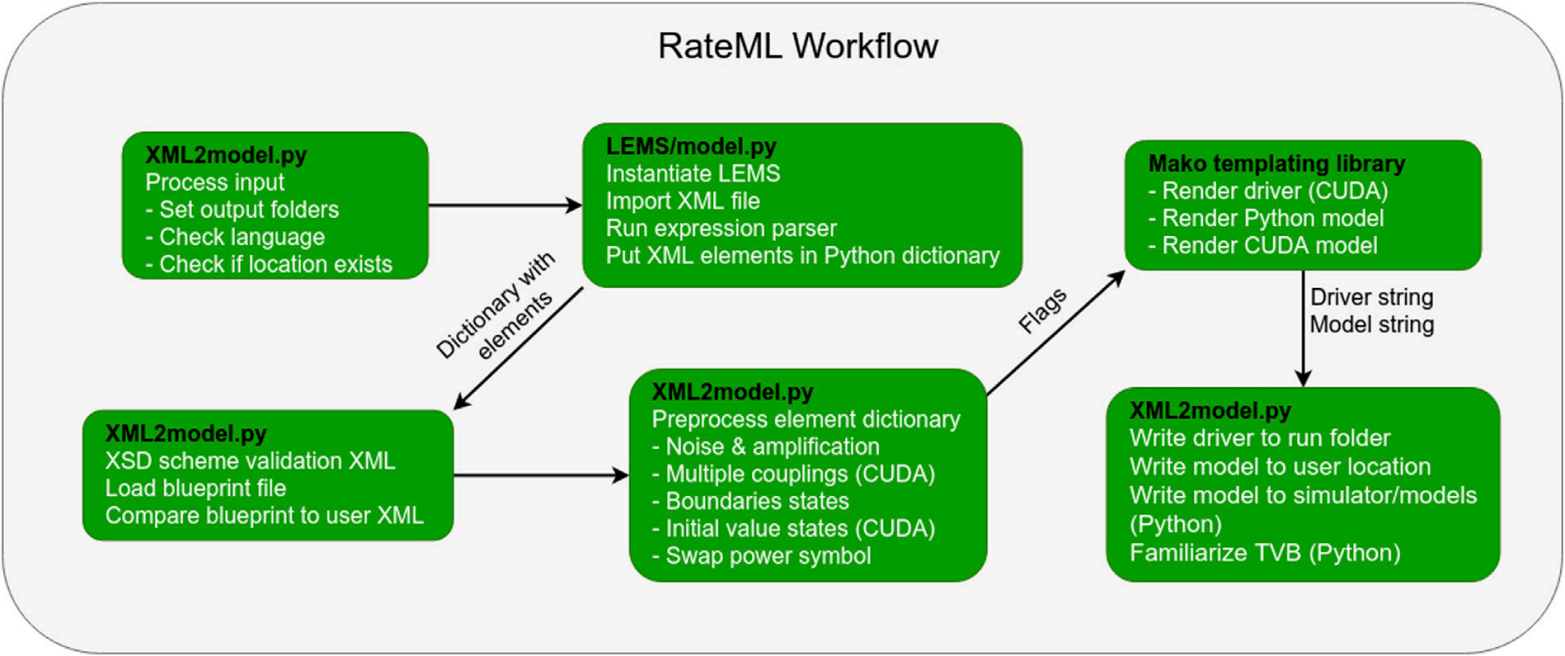
The steps that make up RateML model generation workflow and the software responsible (black text). The flags correspond to the bullets in box 4. The driver and model strings are the translated CUDA model and driver or Python model which only need to be written to file, which happens at the final step.

When the XML file is complete, the NMM can be generated. RateML is part of the TVB main repository; installing TVB through: pip install tvb-library also installs the command line interface operated RateML. To use RateML, a user must import and create an object of the RateML class, enter the arguments model filename, language, XML file location and model output location, and run the code: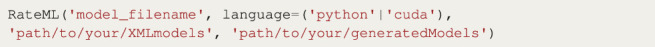 or run from command line:
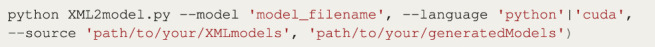


The XML file is converted into a model file using the Mako template engine for Python. When a model generation is started, the flow depicted in Figure 2 is started.

The PyLEMS (Vella et al., 2014) expression parser is used to check and parse mathematical expressions of the XML file. The expression parser embedded in the LEMS library recognises a variety of fundamental mathematical function and operators^2^.

The PyLEMS library returns an expression tree consisting of a large Python dictionary which contains all the aforementioned model components. During the rendering of the model, the elements in the dictionary are projected onto the placeholder expressions of the Mako templates. There are Mako templates for Python, Cuda and the GPU driver module, consisting of dynamic for-loops responsible for generating the code. If a Python conversion is executed, the TVB framework is updated with the latest model. The user can specify a folder in which to save the generated model.

### 3.3 Driver Generation

Generating a model with RateML for CUDA not only produces a model but also a driver file specifically set for that specific model. The driver determines optimal CUDA grid layout, outputs runtime information and makes use of 32 CUDA streams to asynchronously process time steps in combination with memory transfers. The generated driver file model_driver_[model_name]. c is found in the run folder within the RateML folder structure. The fields that dynamically link the model to the driver, are Parameter, DerivedParameter, StatesVariable and Exposure. The number of parameters entered for sweeping together with their ranges and resolution determine the optimal size for the thread grid automatically. The model driver uses the PyCUDA library (Klöckner et al., 2012). To run the model driver, the user must call it in a terminal with the appropriate arguments.


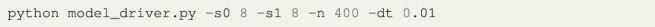


The argument -s*i* refers to the *i*th sweeping parameter defined in the XML file. These arguments set the size of the CUDA grid. In this case a 8 × 8 grid is spawned. If there are more that two parameters that need to be swept, the grid size will adapt and will stay two dimensional. The argument also sets the resolution of parameters, i.e. the number of points in the user defined parameter range. In case of the example of the Kuramoto in Listing 1 the -s0 corresponds to the resolution of global_speed and the -s1 corresponds to global_coupling ranges defined in the derivatives section of the XML file. An argument of size eight returns evenly spaced samples calculated over the range defined in the XML Parameter element. The -n argument sets the number of iteration steps. The -dt argument sets the time step in milliseconds of the integration. More information about the commands can also be found in the documentation in footnote^2^.

**FIGURE 3:**
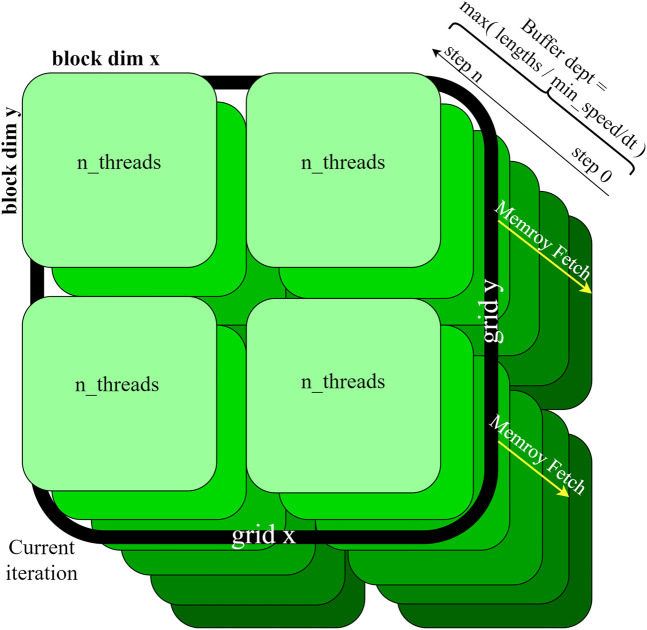
GPU state space specification. Each thread executes a simulation with a unique parameter combination. When the coupling is calculated the kernel steps through all nodes for each time step. At each node, every other node is taken into consideration to calculate the coupling (c.f. Eq 1). The calculated states are stored for as many timesteps back, corresponding to the buffer depth.

The CUDA model spawns a grid in which each thread represents parameter combination of the parameters to be explored (see Figure 3). During the coupling phase, all nodes fetch the states of all other nodes according to the delay specified in the connectivity matrix. This means that the GPU has to store the states of all brain nodes for a certain simulation depth, determined by the largest connection delay in the model. This depth can be configured in the driver software available to drive the CUDA models.

## 4 Use Case: The Montbrió Model

The Montbrió model describes the Ott-Antonsen (Ott and Antonsen, 2008) reduction of an infinite number of all-to-all coupled quadratic integrate-and-fire (QIF) neurons (Montbrió et al., 2015). The two state variables r and V represent the firing rate and the average membrane potential of the QIF neurons. Their derived exact macroscopic equations relate the individual cell’s membrane potential to the firing rate and population mean membrane potential.

This neural mass model was adapted for a study on the role which neuronal cascades play in the causation of whole-brain functional dynamics at rest (Rabuffo et al., 2021). Causality is established by linking structural defined features of a brain network model to neural activation patterns and their variability. The ordinary differential equations that describe the exact firing rates for a network of spiking neurons read:

$${\dot{r}}_{n}\left(t\right)=\Delta /\pi +2r_{n}\left(t\right)V_{n}\left(t\right)+2\sigma \Phi \left(t\right)$$ (2)

$${\dot{V}}_{n}\left(t\right)=V_{n}^{2}\left(t\right)+\eta +Jr_{n}\left(t\right)-\pi^{2}r_{n}^{2}\left(t\right)+I\left(t\right)+4\sigma \Phi \left(t\right)$$ (3)

where *r*_*n*_ and *V*_*n*_ are the firing rate and membrane potential, respectively, of the *n*th neuron, *J* (= 14.5) is the synaptic weight, Δ(= 0.7) is the heterogeneous noise distribution and *η*(= − 4.6) is the average neuronal excitability. Noise enters the equation as Φ(*t*) and its attenuation is *σ*. The attenuation for Φ(*t*) in ${\dot{V}}_{n}\left(t\right)$ is twice as large as in ${\dot{r}}_{n}\left(t\right)$ , this is to keep the relative level of noise the same for both. The coupling term enters as additive current in the average membrane potentials equations, which reads:

$$I\left(t\right)=G\underset{p\neq n}{\sum }W_{np}r_{p}\left(t-\tau_{np}\right)$$ (4)

where the global coupling parameter *G* scales the connectivity matrix *W*_*np*_. The delay is, just as in Eq 1, defined as *τ*_*np*_ = *l*_*np*_/*v*; where the *l*_*np*_ are the lengths between the nodes and *v* is the conduction speed. In contrast to the Kuramoto example; the coupling applied here is not the difference with the current state but is only an accumulation of its connected delayed states. The former is called difference- and the latter is called linear coupling in TVB.

To research the synthetic whole-brain dynamics, Rabuffo et al. (2021) performed a parameter sweep on two global parameters: the global coupling *G* representing the impact of the structure over the local dynamics, and the intensity *σ* which simulates the effect of a generic environmental noise. For each parameterization several minutes of neuroelectric and BOLD activity were simulated.

As a validation test for RateML, we compare the output of the parameter sweep enabled CUDA models against the implementation of the Montbrió model included in TVB. As sweeping over noise would introduce confounding varibility into the results, we substitute a sweep over the global_speed *v* instead of the *σ* parameter, similar to the Kuramoto example above. In the following, we describe how Eqs 2–4 are converted to a CUDA parameter space exploration model using RateML.

### 4.1 Implementing the Model


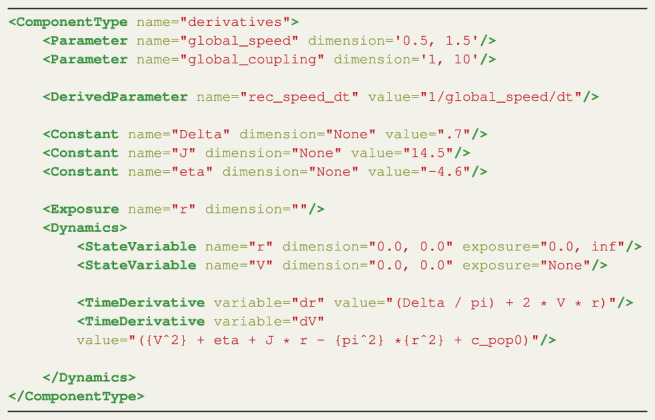


Listing 4 XML derivatives definition for the CUDA Montbrió model.

First the derivative equations are set up. The ComponentType derivatives, shown in Listing 4, details its implementation. As mentioned in the introduction in Section 4, the global_speed and global_coupling are explored; the parameters’ dimension fields set their ranges, which are chosen arbitrarily. The number of points in this range are determined by calling the generated driver file from the command line, see Section 4.2. Next a derived parameter rec_speed_dt is defined, this is identical with TVB’s implementation of the connectome’s conduction speed. The constants match the terms defined in the model equations above; the description fields are omitted here for brevity. Next, the exposure is set to monitor the state variable r.

Both state variables are initialized to 0.0; this can be done by setting the range in the dimension field both to 0.0. Their boundaries are entered in the exposure field. Only the state *r* has a lower bound of 0.0, the upper bound is set to infinity. The TimeDerivative element holds the derivatives which match the differential equations from Section 4. The ComponentType derivatives is followed by the ComponentType coupling, shown in Listing 5.
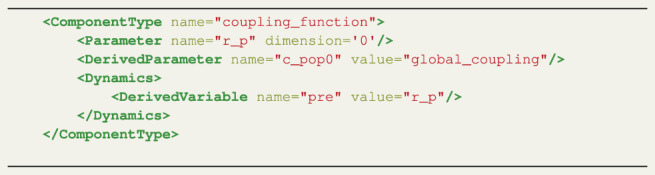


Listing 5 XML Coupling definition for the CUDA Montbrió model.

For the Montbrió model, only the state variable *r* is involved in the coupling calculation, therefore a single coupling function should be defined. First, the Parameter construct defines the coupling variable *r*_*p*_. The coupling function computes the influence of the connected nodes by taking the state values into account. The RateML framework automatically generates code for the coupling function such that the state values of the connected nodes, in this case representing the firing rates, are gathered and processed according to the connection weights. Then, the DerivedParameter construct defines the c_pop0 variable, which links the coupling result to the TimeDerivative element and applies a multiplication with the global_coupling Parameter. The result of which is stored in c_pop0. The defined coupling corresponds to TVB linear coupling meaning that the presynaptic coupling function, specified with the DerivedVariable constructs with the name “pre”, simply holds the coupling variable *r*_*p*_. There is no postsynaptic coupling term, thus a second DerivedVariable can be omitted.

Since no stochastic integration is applied, the XML file should not contain a ComponentType noise.

### 4.2 Validation

A validation setup was used to ensure the accuracy of the results obtained with the automatically generated CUDA code. The validation setup uses the Montbrió model described in the previous section and compares the output to the version of the Montbrió model already present in TVB^3^. This model is sequentially executed for all the parameters using the TVB framework. When RateML is executed to generate the corresponding CUDA model, the produced driver file bears the name model_driver_montbrio.c. A parameter sweep with a grid size of 5*x*10 for 40000 timesteps on a connectome with 68 nodes for *dt* = 0.01 was then setup. The argument to run the generated driver file is as follows:


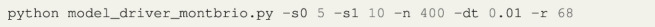


The -s0 and -s1 set the grid to 5*x*10 threads and divide the points evenly for the parameters range, the -dt sets the simulation step time in milliseconds and the -r sets the number of nodes of the connectome. The -n sets the simulation length to 400 resulting in 400/0.01 simulation steps.

The Montbrió model produces a high time-resolution neuroelectric signal, namely the firing rate r and the membrane potential V. Then, a low time-resolution simulated BOLD activity is obtained, by filtering the membrane potentials through the Balloon-Windkessel model. A sliding window approach was applied to obtain the dynamical Functional Connectivity (dFC); inside each time window, a static Functional Connectivity (FC) was computed as the correlation matrix of the BOLD activities. The entries of this windowed-dFC (dFC_*w*_) are defined as the correlation between the FCs at different windows.

**FIGURE 4:**
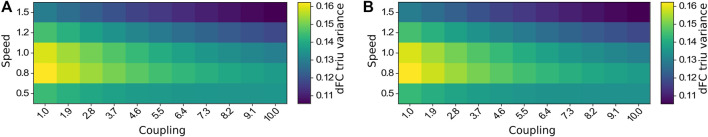
**(A)**. CPU and **(B)**. GPU parameter sweep validation for 68 nodes, 40,000 simulation steps, 0.01 dt on global_speed (*y*-axis) and global_coupling (*x*-axis). The color bar displays the value for the variance of the dFC.

Figure 4 shows the results of parameter simulation validated against the standard Python TVB version. It shows the variance in dFC for each parameter combination. From the figure can be concluded that RateMLs models have similar output. The difference between results is smaller than 13.4*e* ^−5^ ⋅ *t*, relative to the timestep. It shows that the CUDA code is accurate and suited to do parameter sweep experiments.

## 5 Performance

To examine the performance of our approach, we benchmarked three CUDA models, namely the Kuramoto (Kuramoto, 1975), WongWang (Wong and Wang, 2006) and Epileptor (Jirsa et al., 2014), which have one, two and six state variables, respectively. We systematically vary the number of simulated points in the parameter space and integration steps in order to observe the run-time behaviour expressed in iterations per second and memory scaling. The iterations per seconds are calculated as: (*integration*_*steps* ⋅ *parameter*_*space*_*size*)/*elapsed*_*time*.

The benchmark experiments are executed on the JuwelsBooster clusters equipped with A100 GPUs with 40 GB of High Bandwidth Memory two and a bandwidth of 1555 GB/s. In general the GPU’s performance increases, in contrast to a CPU device, by increasing the thread number in combination with the utilization of memory bandwidth; the more threads utilizing memory, the better it can hide the memory latency of the application.

**FIGURE 5:**
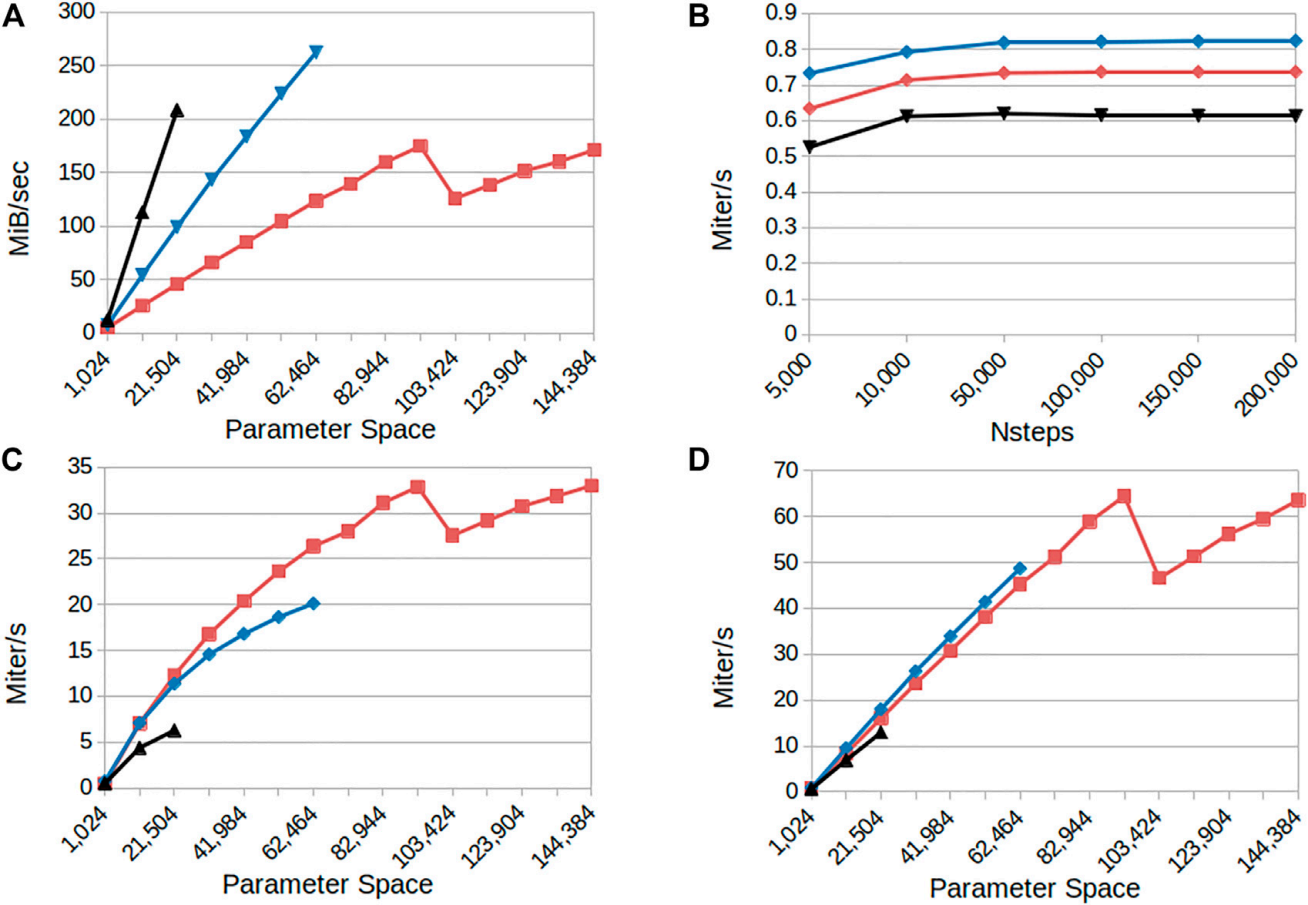
Performance overview for the CUDA parameter sweep models: Kuramoto (red curves), Montbrió (blue curves) and Epileptor (black curves) **(A)** Memory bandwidth consumption as a function of the number of parameter configurations simulated **(B)** Model iterations per second as a function of the number of integration steps (simulated time) for a fixed parameter space size (1,024) **(C)** Model iterations per second as a function of the number of parameter configurations simulated for a small number of integration steps (4,000; 0.4 s simulated time) **(D)** as in **(C)** for 100,000 integration steps (10 s simulated time). Results indicate linear scaling when increasing the parameter space and that the application is memory bound for the higher state models.

The results in Figure 5 panel A show increasing memory bandwidth utilization in relation to a larger parameter space; indicating that the application is memory bound. The Kuramoto, Montbrió and Epileptor models reach their maximum capacities for 40 GB of memory at 144*k*, 62*k* and 22*k* parameters respectively; the plots in panels A, C and D show the results until the memory of the GPU was saturated. The Epileptor reaches its maximum capacity first because it has the most state variables. The parameter capacity is directly related to state size: more states mean more data has to be stored. The single state Kuramoto model has only a single state and has a much larger maximum parameter space. For this model the application reaches it maximum attainable bandwidth at 175 MiB/sec at 91*k* parameters.

Figure 5 panel B shows the behaviour in iterations per second of the RateML generated CUDA models when the simulation length is increased. It shows that there is hardly any increase in the computation speed; for these settings the application is computationally bound. Because the number of iterations cannot be parallelized, there is no increase in memory utilization and hence the GPU cannot increase its performance.

In panel C and D from Figure 5 it is shown that the application performs better when the parameter size increases. The Kuramoto reaches its maximum computation speed at 65 Miters/sec for 91*k* parameters. The executions in panel D for 10s of simulated time reach a higher number of iterations per second than those seen in panel C which ran for 0.4s simulated time. This can be explained from the fact that the GPU uses 32 streams to asynchronously copy data from the GPU back to disk, which is usually the most time consuming part of the application. The memory copying can be masked by asynchronously starting a new integration step in a new stream, in parallel to the memory movement. When a GPU instance is still busy with copying data to disk another stream can start the next integration step. The more simulation steps performed the larger the benefit from asynchronously copying data to disk, resulting in an increase in the iterations per second.

**FIGURE 6:**
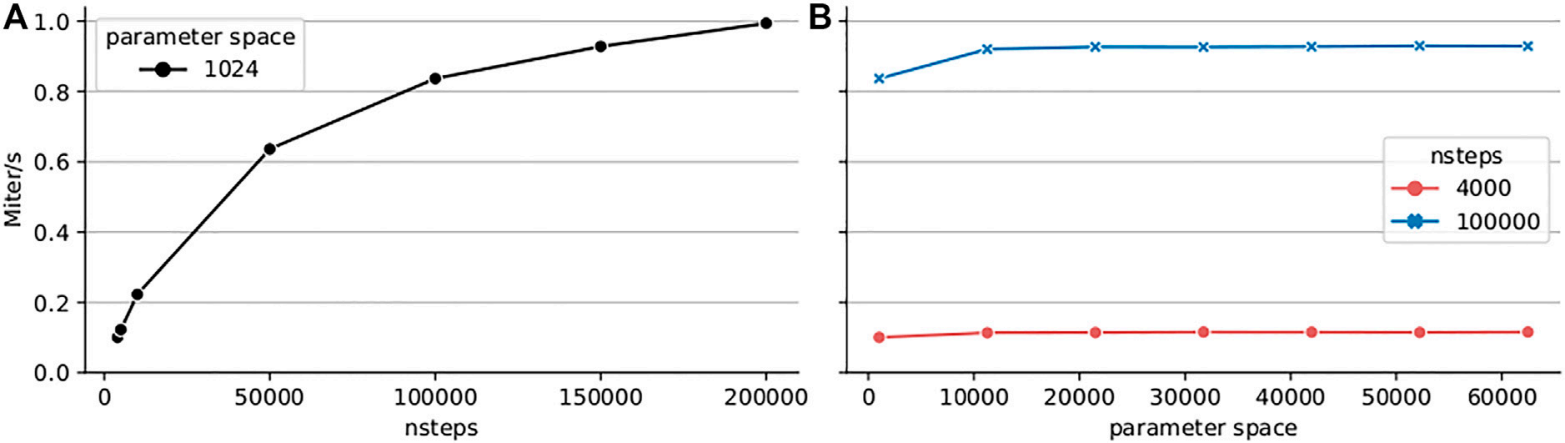
Performance of the code-generated TVB Numba backend (CPU) on a single Cray XC40 node (2×18 cores, 2.10 GHz, 128 GB RAM). Execution time of a parameter space exploration was measured for the Montbrió model on a connectome of 68 nodes for **(A)** fixed parameter space size (1,024 points) and varying number of integration steps, **(B)** small number of integration steps (4,000) corresponding to 0.4 s of simulated time, and longer simulation of 100,000 or 10 s of simulated time.

Finally, for reference we present current performance of the Python TVB library using the Numba backend (Figure 6). For this we have configured the simulation with the Montbrió model driven by noise and a connectome of 68 nodes and delays induced by a propagation speed of 2 m/s. The benchmark was executed on a single Cray XC40 node (Intel Xeon E5 − 2,695 v4, 2 × 18 cores, 2.10 GHz, 128 GB RAM) of the multicore partition located in Piz Daint. The execution of different parameters was orchestrated by the multiprocessing Python package using all 36 cores of the compute node.

The results presented in Figure 6A show that the performance increases with the length of the simulation suggesting better amortization of the simulation initialization overhead and better data locality. We explored two simulation lengths with this model: a 4 000 iteration step microbenchmark and a longer experiment of 100000 integration steps corresponding to 10 s of simulated time which spans several BOLD time points (usual sampling frequency 1*Hz*) and is more representative of real workloads. The results presented in Figure 6B indicate that the peak performance is already reached for small parameter space sizes. Longer simulations achieve better performance due to amortization of simulation launch overhead and increased data locality. For a particular simulation length, the peak performance is reached already for small parameter spaces.

## 6 Discussion

In this work we presented RateML, a model code generator based on LEMS for defining neural mass models succinctly. TVB simulations vary greatly in simulation time, number of nodes used and which parameters to explore within the different research topics and science groups. One thing is clear, in order to have the simulation results fit subject data for clinical research or have an optimal resolution for, e.g., the cohort studies for aging, the number of parameters that need to be explored are vast; and it is safe to say that the more parameters explored the better. RateML enables the user to generate complex Python and CUDA neural mass models and to do fast parameters sweeps on the GPU, with identical results as when done with TVB. We introduced the interface and its elements, the inner workings of the generator and the relation of the elements to the placeholder templates which generate the code for neural mass models and the GPU driver. At the moment some of the variables within the LEMS components used in RateML have been modified to fit specific functionality and match the TVB simulation strategy. Even though at the moment models produced by RateML can not be directly ported to other simulators which support LEMS and are able to simulate BNMs or NMMs, work is being done in collaboration with the LEMS development community to fit all the requirements and perform any required modifications to RateML or extensions to the standard.

In the use case section we demonstrated how to define the XML file for the Montbrió model, generate the CUDA code and driver, and do fast and efficient parameter sweeps over the global speed and global coupling parameters using a GPU. Several other models are already currently available in XML format at the TVB repository. We showed that the output is very close to the TVB simulator. This verifies that the code generation process is faithful to the reference implementation in TVB. For simplicity and clarity in the validation procedure, noise has been neglected from these tests. However, RateML can include noise within the model description.

We have benchmarked the code and shown that the GPU version is memory bound and the runtime for the models generated with RateML scales linearly to the number of explored parameters, up to 144,000 parameters on a single GPU. Results also show that the GPU performs even better with larger parameter spaces, due to the fact that it can then hide memory latency more effectively. In Figure 5 it is also visible that the amount of iterations per second in the GPU continues to increase as the number of parameter combinations grows. This translates into a progressive utilization of the GPU computing resources and almost constant simulation times for any amount of parameters which can fit in memory. The application could benefit from a multi GPU setup enabling users to do even larger parameters sweeps.

## 7 Conclusion and Future Work

While the work on performant code generation in RateML has focused on NVIDIA GPUs, because of their prevalence in HPC centers, future work in RateML will address better code generation for CPUs, from generating full simulations for CPU instead of just the model derivatives and ensuring Single Instruction Multiple Data (SIMD)^4^ instruction sets are correctly used, instead of relying on Low Level Virtual Machine (LLVM) (Lattner and Adve, 2004) autovectorization, to sizing parameter space exploration work arrays to stay within CPU level 3 cache. These improvements allow for end users to do significant interactive prototyping prior to sending a model for full sweeps or optimization.

To further streamline computational workflows involving brain atlas data, RateML will add data specifications, which enable fetching the requested data from an online atlas, transformed into a model on which a parameter sweep is performed to fit the model against, e.g. the fMRI belonging to the requested subject’s brain. This will also include integrating new atlas data on per-region variability, e.g. receptor densities, and mapping these to model parameters. Deep integration between multimodal neuroimaging data, anatomical data, and high-performance code generation is a key unique feature enabling non-expert users to take advantage of neural mass modeling techniques.

For workflows that involve extensive parameter tuning, it is frequently required to use adaptive algorithms and not rely on full grid explorations. This is specialy important where the dimensionality of the parameter space is high. For instance, to optimize resource utilization one could make use of stochastic gradient descent or other algorithms which selectively focus on regions of interest within the parameter space. While RateML can scale models to tens of thousands of parameter combinations, hyper-parameter optimization can be delegated to a framework such as Learning to Learn (L2L) (Subramoney et al., 2019). This framework is a hyper parameter optimizing network consisting of two loops, an inner loop which handles the process to be optimized and an outer loop which handles the hyper parameter optimization. The user can instantiate many optimizer algorithms such as gradient descent, evolutionary strategies or cross-entropy to find the best fitting algorithm for each use case. With RateML, which acts as a front-end to this framework, the user is able to generate a parameter sweep-enabled model and directly run it, as a multi agent optimizee within the L2L framework, enabling new use cases with complex hierarchical models.

In summary, RateML provides the neuroscience community with a new tool to easily define, generate, and simulate BNMs and NMMs. The high throughput of simulation results which can be derived of this frameworks helps match the increasing production of data by experimental neuroscientists and the equal need for its processing, analysis and interpretation. By simplifying the access to state of the art computing methods, RateML enables the further understanding of the brain function merging dynamical models based on experimental data and fast parameter exploration.

## Data Availability

The raw data supporting the conclusion of this article will be made available by the authors, without undue reservation.

## Footnotes

1
https://github.com/the-virtual-brain/tvb-root.git.

2
https://github.com/the-virtual-brain/tvb-root/blob/master/scientific_library/tvb/rateML/README.md for further documentation.

3
https://github.com/the-virtual-brain/tvb-root/blob/master/scientific_library/tvb/simulator/models/infinite_theta.py.

4
https://www.sciencedirect.com/topics/computer-science/single-instruction-multiple-data.
